# Commentary: Brain, Mind, World: Predictive Coding, Neo-Kantianism, and Transcendental Idealism

**DOI:** 10.3389/fpsyg.2017.02077

**Published:** 2017-12-05

**Authors:** Michał Piekarski

**Affiliations:** Institute of Philosophy, Cardinal Stefan Wyszyński University in Warsaw, Warsaw, Poland

**Keywords:** predictive processing, representationalism, prediction, anticipation, normativity

Zahavi claims that the predictive processing (PP) approach supports a radical neuro-representationalism, “according to which the content of our conscious experiences is a neural construct, a brain-generated simulation” (Zahavi, 2017, p. 1). He claims that this is because, on the one hand, spontaneous human cognition becomes equated with the model of cognition developed by sciences, and on the other, the representational position challenges the belief concerning the objectivity of the world of experience. It is of key importance, therefore, to answer the question posed in the article: “Which position is the best able to accommodate our natural inclination for realism: Contemporary neuro-representationalism or Husserl's transcendental idealism?” (Zahavi, 2017, p. 2). The author defends the answer that refers back to Husserl's thought.

In this commentary, I will demonstrate that (1) the understanding of PP as suggested by Zahavi is not adequate for the different accounts offered in the literature; (2) we can identify such PP conceptions which do not continue neo-Kantism; and (3) the problem of the normativity of prediction enables us to treat PP and Husserl's phenomenology as complementary rather than antagonistic approaches.

## Many faces of predictive processing

Zahavi develops the PP conception mainly on the basis of the works by Hohwy, Metzinger and Frith focusing on the so-called neurocentricism (see Wachowski, 2014). Nonetheless, Hohwy's publications, so very important for the author of the article, are not representative of the entire movement deriving from the idea of PP. Hohwy's strong representationalism is just one possible position (Bruineberg, 2017; Dolega, 2017). It is linked to the so-called Conservative PP (Hohwy, 2013; Gładziejewski, 2016). The proponents of the more Radical PP (Clark, 2013, 2015a, 2016; Orlandi, 2016, 2017) emphasize that action and perception stand in close relation to each other. They also claim that some levels of the hierarchical generative model are directly representational, whilst others are only indirectly so, being related to the world in an enactive way, which means that representations “aim (is) to *engage* the world, rather than to depict it in some action-neutral fashion” (Clark, 2015b, p. 4). In addition, Clark acknowledges the existence of the Really Radical PP (Clark, 2015c) where the concept of representation is abandoned altogether to the benefit of the dynamic approach.

It follows that the critique of PP made by Zahavi concerns only propositions of a few authors and is not relevant to the conception as a whole.

One also has doubts whether the simple division into idealism/realism should be applied to PP. Gładziejewski (2017), for example, maintains representationalism but demonstrates that receptivity of sensory input guarantees direct access to the world.

## Predictive processing and ecological approach

The other objection Zahavi has against the PP approach is its neo-Kantianism. However, Kantianism is not the only tradition in which the idea of PP is rooted. A number of authors have stressed that the concept is dependent on Gibson's ecological psychology and his belief about our direct cognitive access to the world (e.g., Bruineberg et al., 2016; Orlandi, 2016, 2017; Bruineberg, 2017). Orlandi justifies that seeing is not inferential but embedded into the biological structure of the organisms. For Bruinberg (inspired by Merleau-Ponty), PP is about steering interactions with the environment is such a way that the cognitive system understood as the agent could develop fully in an adequate environment.

The belief about the neo-Kantian “burden” of the PP approach goes hand in hand with the conviction about its strong representationalism.

## Prediction, phenomenological anticipation, normativity

According to the thesis advanced in the article, the PP approach should be contrasted with Husserl's phenomenology. In spite of Zahavi's assertions, however, it seems that there is a way to link the perspectives of PP and phenomenology. We can achieve this by analyzing the role played by predictions in PP and anticipations in phenomenology.

Late Husserl emphasized that perception is a process based on (1) the experience of objects given in a possible intuition, and (2) anticipations of possible bodily actions in the world which are later realized by relevant actions (Madary, 2016). Such an approach corresponds to the fundamental PP idea that perception is an active and dynamic process unfolding in the environment: a generative model developed top-down tries to anticipate the flow of subsequent sequences of bottom-up sensory data. The category of prediction/anticipation is crucial for both concepts as the content of predictions/anticipations determines the actions taken by the agent. Predictions/anticipations serve a normative function because they oblige the agent to take some action (Friston, 2010, p. 233) which is strictly connected with an “active inference” in the neurobiology literature.

In phenomenology, all experiences are governed by anticipations of normality which consist in transposing some information generated by previous experiences onto the present experience (Husserl, 1966, p. 186). On the one hand, it is important objectively as relating to all potential experiences of the object of sensory modalities. On the other, it has normative significance because it is thanks to such information that a given experience is felt as objectively important, that is obliging the subject to some cognitive and non-cognitive actions.

In the PP approach, the normative nature of predictions should be linked with active inference (Friston, 2010, p. 129) consisting in such interventions/actions in the world that help to uphold a given hypothesis formulated by the model. We should bear in mind that the reduction of the predictive error in the hierarchical generative model implies a “low level” biological normativity which has to do with keeping a living system in a condition far from thermodynamic balance. Friston calls it the *free-energy principle*. On higher levels of the generative model, normativity is related to:

1. patterns of neuronal excitations based on predictions;
2. the role played by predictions in decision-making, emotional and action control processes, among others (Piekarski, 2017, Piekarski, in preparation).

The analyses presented herein require detailed development and justification. Even in their present form, however, they already pave the way for outlining a creative dialogue between phenomenology and the PP framework. Consequently, they challenge Zahavi's thesis whereby these two positions are separate.

## Author contributions

MP reviewed the literature, developed the theoretical stance, wrote the manuscript and prepared to publication.

### Conflict of interest statement

The author declares that the research was conducted in the absence of any commercial or financial relationships that could be construed as a potential conflict of interest.
